# Implications of the USP10-HDAC6 axis in lung cancer - A path to precision medicine

**DOI:** 10.46439/cancerbiology.2.015

**Published:** 2016-04-22

**Authors:** Xiaohong Mary Zhang, Navnath Gavande, Prahlad Parajuli, Gerold Bepler

**Affiliations:** 1 Department of Oncology, Wayne State University School of Medicine, Karmanos Cancer Institute, 4100 John R. Street, Detroit, Michigan, 48201, USA; 2 Department Pharmaceutical Sciences, Eugene Applebaum College of Pharmacy and Health Sciences, Wayne State University, 259 Mack Avenue, Detroit, Michigan, 48201, USA

## Abstract

Lung cancer is the leading cause of cancer death among both men and women in the United States. Because lung cancer is genetically heterogeneous, tailored therapy alone or in combination with chemotherapy would increase patient overall survival as compared with the one-size-fits-all chemotherapy. *TP53*-mutant lung cancer accounts for more than half of all lung cancer cases and is oftentimes more aggressive and resistant to chemotherapy. Directly targeting mutant p53 has not yet been successful, so identification of novel therapy targets and biomarkers in the *TP53*-mutant lung cancer is urgently needed to increase the overall survival in this subgroup. Deubiquitinating enzymes (DUBs) regulate a vast majority of proteins (DUBs’ substrates) via removal of ubiquitin moieties or ubiquitin chains from these proteins, thereby altering the stability and/or functions of these substrates. In this review, we will focus on a DUB, referred to as ubiquitin-specific peptidase 10 (USP10) whose substrates include both oncogenic proteins and tumor suppressors. Therefore, targeting USP10 in cancer is highly context-dependent. Here, we will discuss USP10’s functions in cancer by examining its various known substrates. In particular, we will elaborate our recent findings in the oncogenic role of USP10 in the *TP53*-mutant subgroup of lung cancer, focusing on USP10’s function in the DNA damage response (DDR) via histone deacetylase 6 (HDAC6). Overall, these findings support the notion that targeting USP10 in the *TP53*-mutant subgroup of NSCLC would sensitize patients to cisplatin-based chemotherapy. Generating potent and specific clinically relevant USP10 inhibitors would benefit the *TP53*-mutant subgroup of NSCLC patients.

## Introduction

Post-translational modifications (PTMs) participate in virtually all biological processes; as such, the enzymes responsible for these PTMs are pivotal for normal cellular functions. One of these PTMs, ubiquitination, is regarded as a key regulator of a myriad of complex cellular processes; it may rival phosphorylation in scope and exceed it in complexity. As a reversible PTM, ubiquitination is governed by two groups of enzymes that either attach or remove the modification. The first group is responsible for adding the ubiquitin moieties or polyubiquitin chains to the target proteins or substrates. In doing so, three types of enzymes—E1 ubiquitin-activating enzymes, E2 ubiquitin-conjugating enzymes, and E3 ubiquitin ligases—are required to coordinate the completion of this task [1]. The second group, deubiquitinating enzymes (DUBs), catalyze the reverse reaction—removing the ubiquitin moieties or polyubiquitin chains from the substrates [2]. One of the major functions of E3 ubiquitin ligases and DUBs is governing protein homeostasis— the former promotes protein degradation mainly via the ubiquitin-proteasome pathway, and the latter stabilize proteins via removal of the polyubiquitin chains from the target proteins. The human genome encodes ~600 E3 ubiquitin ligases and ~100 DUBs [3]. Mounting evidence indicates that dysregulation or aberrant expression of E3 ubiquitin ligases or DUBs results in the development of a plethora of diseases, including cancer [4]. Based on their catalytic mechanism, DUBs fall into two groups [3]. The first group utilizes the cysteine amino acid in the catalytic triad, hence why these DUBs are referred to as thiol proteases or cysteine proteases. They can be further classified into five families: ubiquitin-specific proteases (USPs), ubiquitin C-terminal hydrolases (UCHs), ovarian tumor proteases (OTUs), Machado-Joseph disease proteases (MJD), and the newly identified motif interacting with Ub-containing novel DUB family proteases (MINDYs) [3]. The second group, whose activation requires a zinc ion, only contains one family: JAB1/MPN/Mov34 metalloenzymes (JAMMs) [2].

USP10 belongs to the USP family, which consists of 54 members and is the largest family in the DUB superfamily [5]. USP10’s role in cancer has been intensively studied in the past decade. Numerous tumor suppressors and oncogenic proteins have surfaced as USP10’s substrates [6–17], and the list of these substrates is still rapidly growing (Table 1). Of note, the most famous tumor suppressor, p53 protein, was identified as USP10’s substrate in 2010 [6]. Therefore, it is conceivable to imagine that like p53 USP10 is a tumor suppressor as well, since USP10 stabilizes p53 to enhance p53’s tumor suppressor function. However, as a DUB, USP10 may also regulate the stability of mutant p53 (mutp53) and USP10 could serve an oncogenic role in *TP53*-mutant cancers. In an indirect scenario, USP10 could either exacerbate or dampen the oncogenic function of mutant p53 through its substrates. In support of the second scenario, we recently discovered that the depletion or inhibition of USP10 in *TP53*-null or *TP53*-mutant non-small cell lung cancer (NSCLC) reduces tumor burden in a xenograft mouse model and sensitizes tumors to cisplatin, highlighting USP10’s chemo-resistant role in this subset of NSCLC. Mechanistically, we have shown that USP10 regulates the DNA damage response (DDR) via histone deacetylase 6 (HDAC6) to confer cisplatin resistance, as HDAC6 regulates multiple DDR proteins. In this review, we will further discuss the above findings and ask the following open question: Why USP10 functions differently in *TP53*-wild-type and *TP53*-mutant subgroups of lung cancer?

### USP10’s Function Differs in Wild-Type TP53 and Mutant TP53 NSCLC Cell Lines

In 2014, we reported that HDAC6 serves as a ubiquitin E3 ligase to promote degradation of a key DNA mismatch repair protein MSH2 [18]. Logically, we were curious about the enzyme, which counteracts HDAC6 to stabilize MSH2 and employed a protein purification approach to examine the proteins associated with MSH2. We immuno-precipitated MSH2 and identified MSH2-associated proteins by mass spectrometry analysis (LC-MS/MS). As expected, we found that most of the peptides detected in the MSH2 complex belong to MSH6—a well-known binding partner of MSH2—which forms a heterodimer with MSH2 referred to as MutSα [19] Interestingly, we indeed found that abundant peptides in the complex belong to a DUB, USP10. We then set out to investigate whether USP10 stabilizes MSH2, leading to an increased level of the MSH2-MSH6 complex (MutSα), which could sense various DNA adducts and trigger the downstream signaling to promote apoptosis or cell cycle arrest. We published our findings in 2016 showing that the depletion of USP10 confers cellular resistance to both drugs, 6-TG (6-thioganuine) and MNNG (N-methyl-N’nitro-N-nitrosoguanidine), in NSCLC cells due to the decreased level of MSH2 [20]. As we further tested the role of USP10 in cisplatin sensitivity in a large panel of NSCLC cell lines, surprisingly, we discovered that upon cisplatin treatment the depletion or inhibition of USP10 could lead to two entirely different outcomes: cell survival or apoptosis, depending on the cellular status of *TP53*—USP10 promotes cisplatin sensitivity in wild-type p53 cells, while USP10 confers cisplatin resistance in mutant p53 cells. This unexpected finding prompted us to study the underlying mechanism by which USP10 confers cisplatin resistance in mutant p53 cells.

### The Role of HDAC6 in Cancer and the DNA Damage Response

HDAC6 belongs to the class IIb HDAC family and is a well-known oncogenic protein [21,22]. HDAC6 is overexpressed in numerous cancers, including gastric cancer [23], glioblastoma [24], and melanoma [25]. Our current publication has shown that HDAC6 protein is overexpressed in all three histological subtypes of NSCLC patient samples-adenocarcinoma, squamous cell carcinoma, and large cell carcinoma [26]-suggesting the oncogenic role of HDAC6 in NSCLC. Recently, the role of HDAC6 in chemotherapy efficacy in several cancers, including lung cancer, has been explored by our group and others [27,28]. For example, HDAC6 confers resistance to temozolomide in glioblastoma [24], and the HDAC6-selective inhibitor ACY-1215 accelerates vemurafenib-induced cell death of BRAF-mutant melanoma cells [25]. Our group is among the first to show that HDAC6 is associated with cisplatin resistance in NSCLC cells [28]. However, the mechanism by which HDAC6 confers chemo-resistance is not fully revealed.

HDAC6 has been well-established as a master regulator of cellular stress responses, including ubiquitinated/misfolded protein stress [29], ER stress [25], and genotoxic stress [27,28]. Our group is a pioneer in defining the role of HDAC6 in the DDR. Structurally, HDAC6 is unique among the members of the HDAC family in that it contains two tandem repeats of deacetylase domains (referred to as DAC1 and DAC2), an SE14 motif [30], and a ZnF-UBP domain [31] that binds to mono-, poly-ubiquitin chains and ubiquitinated proteins. We are the first to reveal that DAC1 harbors ubiquitin E3 ligase activity toward a key DNA mismatch repair protein, MSH2, thereby regulating the homeostasis of MutSα and the DDR [18]. Our follow-up study has revealed that HDAC6 could also deacetylate MLH1 and decrease the formation of the MutSα -Mutα complex, leading to 6-TG resistance [32]. Most recently, we reported that HDAC6 ubiquitinates a crucial cell cycle checkpoint kinase 1 (Chk1) and confers radioresistance in NSCLC [33]. Because the DNA mismatch repair (MMR) complex (MuSα-MutLα) interacts with the protein components in the ATR-Chk1 pathway, we hypothesize that HDAC6 governs DDR via the MMR complex as well as the ATR-Chk1 signaling. As aforementioned both HDAC6 and USP10 regulate MSH2, we then tested whether USP10 and HDAC6 interact. Our investigations then revealed that HDAC6 interacts with USP10, and it is a substrate of USP10. Therefore, we suspect that USP10 regulates DDR via HDAC6.

### The USP10-HDAC6 Axis in a mutp53 Background

**Figure 1:**
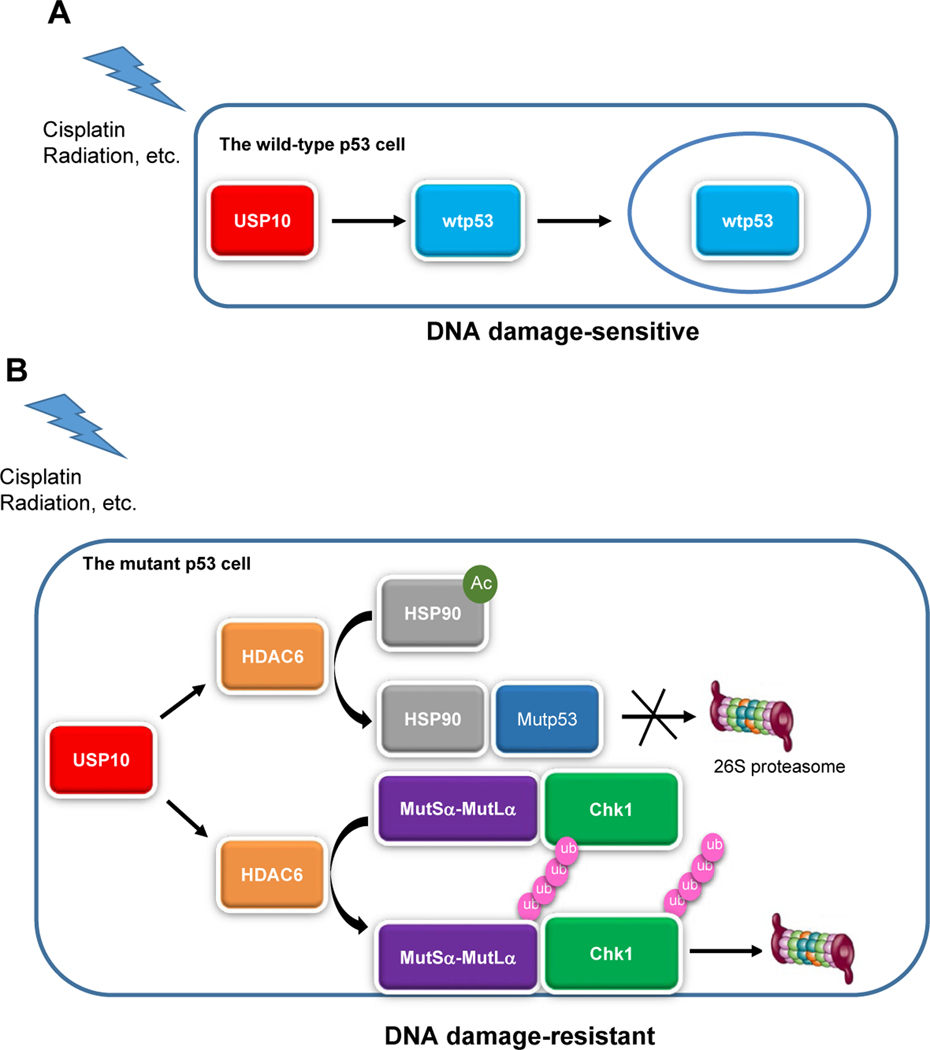
USP10 functions distinctly in wild-type p53 and mutant p53 lung cancer cells upon DNA damage. A**)** In wild-type p53 cells, USP10 stabilizes and translocates p53 in the nucleus and sensitizes cells to DNA damage, such as ionizing radiation, **B)** whereas in the mutant p53 cells, USP10 stabilizes HDAC6. There are two ways HDAC6 could then contribute to chemo- and radio- resistance: HDAC6 exerts its deacetylase activity to deacetylate HSP90 and promote binding to mutant p53, leading to hyperstability of mutant p53. Or HDAC6 exerts its ubiquitin E3 ligase activity and deacetylase activity to promote degradation of the DNA mismatch repair complex (MutSα-MutLα) and cell cycle checkpoint kinase 1 (Chk1), contributing to chemo- and radio- resistance. Our model suggests that developing USP10 inhibitors will benefit lung cancer patients harboring mutant p53.

In general, mutp53 proteins promote cancer cell proliferation by acting as homeostatic factors that sense and protect cancer cells from transformation-related stress stimuli, including DNA lesions, oxidative and proteotoxic stress, metabolic imbalance, interaction with the tumor microenvironment, and the immune system [34]. Molecules, which dampen mutp53’s oncogenic function, could be exploited as targets for cancer treatment. Previous investigation has shown that HDAC6 deacetylates HSP90, a heat shock protein, which stabilizes mutp53 [35]. Therefore, USP10 could contribute to mutp53 stabilization via stabilizing HDAC6. However, it is unclear whether USP10 could also directly stabilize mutp53. Thus, we suspect that USP10 could reduce the DDR and stabilize mutp53 to promote tumor progression, and confers cisplatin resistance via HDAC6 in the mutp53 subset of NSCLC (Figure 1).

### Perspectives

As both USP10 and HDAC6 are enzymes and each has numerous substrates, using proteomics approaches to identify their substrates would provide a big picture of their roles in NSCLC. In addition, since both could play roles in the nucleus, genomics studies on USP10 and HDAC6 would provide evidence on how they affect gene transcription. Overall, we will gain a better understanding of the USP10-HDAC6 axis by an integrated genomics and proteomics approach. Translationally, developing potent and specific USP10 inhibitors with medicinal chemists would further establish USP10 as an important target in the mutp53 subset of lung cancer. Finally, as the current landscape for the treatment of advanced lung cancer has shifted towards anti-PD-l/PD-L1-based immunotherapy [36], further investigations to connect the USP10-HDAC6 axis to immunotherapy are certainly warranted.

## Figures and Tables

**Table 1:** USP10 substrates.

Tumor suppressors	Cancer types	References
p53	Renal cell carcinoma	Yuan et al., 2010 [6]
PTEN	Lung cancer	Sun et al., 2018 [7]
P14ARF	Non-small cell lung cancer	Ko et al., 2018 [8]
KLF4	Lung cancer	Wang et al., 2020 [9]
SirT6	Colon cancer	Lin et al., 2013 [10]
**Oncogenic proteins**		
Musahi 2	Colon cancer	Ouyang et al., 2019 [11]
Raf-MEK-ERK	Endometrial cancer	Chen et al., 2018 [12]
FLT3-ITD	Acute myeloid leukaemia	Weisberg et al., 2017 [13]
Spleen tyrosine kinase (SYK)	Acute myeloid leukaemia	Yang et al., 2020 [16]
G3BP2	Prostate cancer	Takayama et al., 2018 [14]
Slug/Snail2	Epithelial-mesenchymal transition	Ouchida et al., 2018 [15]
YAP/TAZ	Liver cancer	Zhu et al., 2020 [17]
HDAC6	Non-small cell lung cancer	Hu et al., 2020 [26]
